# A new striking and critically endangered species of *Nasa* (Loasaceae, Cornales) from North Peru

**DOI:** 10.3897/phytokeys.121.33927

**Published:** 2019-04-24

**Authors:** Tilo Henning, Rafael Acuña Castillo, Eric Frank Rodríguez Rodríguez, Luis Felipe García Llatas, Maximilian Weigend

**Affiliations:** 1 Freie Universität Berlin, Botanischer Garten Botanisches Museum, Königin-Luise Str. 6-8, 14195 Berlin, Germany Freie Universität Berlin Berlin Germany; 2 Nees Institut für Biodiversität der Pflanzen, Rheinische Friedrich-Wilhelms-Universität Bonn, Meckenheimer Allee 170, 53115, Bonn, Germany Rheinische Friedrich-Wilhelms-Universität Bonn Germany; 3 Universidad de Costa Rica, Escuela de Biología, Apdo. Postal: 11501-2060 San Pedro de Montes de Oca, Costa Rica Universidad de Costa Rica San Pedro de Montes de Oca Costa Rica; 4 Herbarium Truxillense (HUT), Universidad Nacional de Trujillo, Jr. San Martín 392, Trujillo, Perú Universidad Nacional de Trujillo Trujillo Peru; 5 Servicio Nacional de Áreas Naturales Protegidas por el Estado (SERNANP), Calle Diecisiete 355, San Isidro-Lima, Perú Servicio Nacional de Áreas Naturales Protegidas por el Estado Lima Peru

**Keywords:** Loasaceae, Peru, Laquipampa, *
Nasa
*, Lambayeque, Amotape-Huancabamba-Zone, narrow-endemic, Chiñama, *
Angeldiazia
*, new species

## Abstract

*Nasaangeldiazioides***sp. nov.** is described and illustrated. The species is restricted to two forest remnants on the western slope of the northern Peruvian Andes (Dept. Lambayeque) where it is found in the undergrowth of primary forest. The new taxon shows a unique leaf morphology in the family Loasaceae. Molecular and morphological data show that the new species belongs to the *Nasatriphylla* group. Since the relic forests of the north-western Andes are increasingly threatened by the effects of climate change, i.e. droughts and wildfires, the new species already faces imminent extinction.

## Abstract (es)

Se describe e ilustra la especie inédita *Nasaangeldiazioides***sp. nov.** Esta especie está restringida a dos remanentes de bosque de la vertiente occidental de los Andes del norte del Perú (Depto. Lambayeque), donde se la encuentra en el sotobosque de bosques primarios. *Nasaangeldiazioides* muestra una morfología foliar única en la familia Loasaceae. Tanto la evidencia morfológica como la molecular muestran que esta nueva especie pertenece al grupo de *Nasatriphylla*. Dado que los bosques relictos de los Andes noroccidentales del Perú se encuentran cada vez más amenazados por los efectos del cambio climático, como lo son las sequías e incendios forestales más frecuentes, esta nueva especie podría estar enfrentando una extinción inminente.

## Introduction

Loasaceae Juss. and its largest genus *Nasa* Weigend (97 spp.) constitute a prime example for the challenges botany faces today when trying to assess Andean phytodiversity. *Nasa* has been recognised as the most speciose genus in the family since its segregation from *Loasa* Adans. (Weigend 1997, 2006; Weigend et al. 2006a) and as the result of ongoing taxonomic and systematic research based on numerous field collections and the study of extensive herbarium material. Almost two thirds of the species and numerous subspecies were described during the last two decades (Weigend 2000a, b, 2001, 2004; Rodríguez 2008; Henning and Weigend 2011; Henning et al. 2011). The increasingly longer intervals between new discoveries suggest that the majority of suitable habitats have been sampled and that the alpha-taxonomy may be approaching completion. However, this taxonomic research, fundamental for every conservation effort, has become a race against time in recent years. Unfortunately, botanical sampling, as the crucial basis for subsequent conservation of Andean biotopes – even if funding is available – usually follows the expanding human activities throughout most of Latin America and across all relevant altitudinal levels (Feeley and Silman 2011; Oliveira et al. 2016). Agricultural and mining activities, as well as urban sprawl, have become an immense threat, especially for the hyperdiverse mosaic landscapes and habitats within the Andes and their biota (Weigend et al. 2005, 2006b). Moreover, in recent years, the impact of climate change has been strongly affecting the region and, amongst other effects, has led to severe droughts, even in evergreen high Andean cloud forest, resulting in tremendous wildfires (Mutke et al. 2017) that destroyed numerous forest remnants, especially on the western slopes of the Andes. The relic forests in this area are home to a number of micro-endemics (Weigend et al. 2005, 2006b), many of which would be potentially extinct or critically endangered (Rodríguez and Weigend 2006). An unknown number of taxa awaits formal scientific description, as is the case in other biodiversity hotspots worldwide (Joppa et al. 2011).

**Figure 1.**
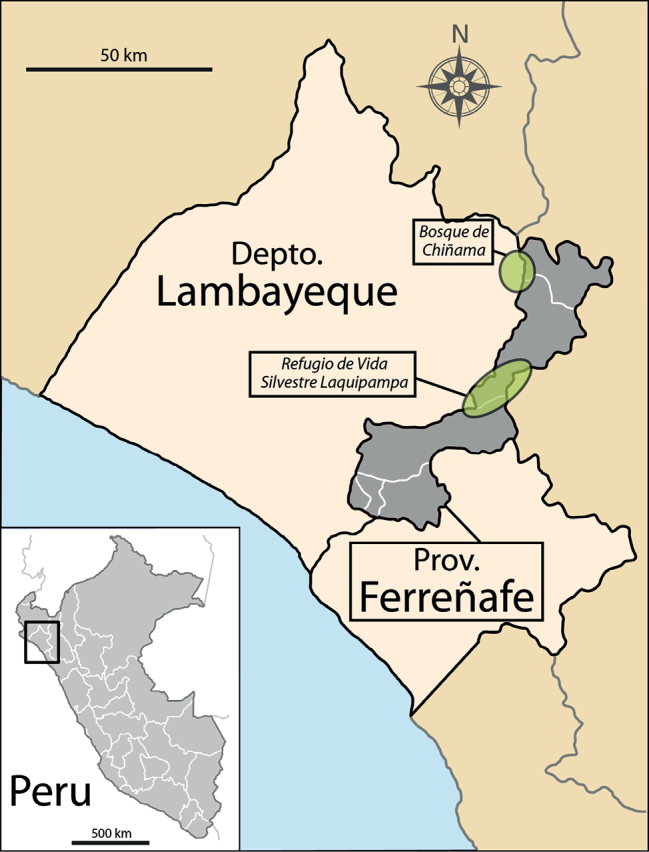
Map of the known localities of *Nasaangeldiazioides*.

The latest taxonomic additions to Loasaceae in general and *Nasa* in particular, have consequently either been discovered in remote areas that were botanically sampled only recently, often following new road cuttings (e.g. *Nasatulipadiaboli* T.Henning & Weigend, three subspecies of *Nasarugosa* Killip, Henning et al. 2011; *Nasakuelapensis* Weigend, Weigend and Rodríguez 2003) or have been revealed by evaluation of herbarium material from poorly sampled localities (e.g. *Nasapascoensis* Weigend, Weigend 2000b, *Nasacallacallensis* Weigend & E.Rodr. Weigend and Rodríguez 2002). Conversely, the taxon here described has been discovered in a relatively well known and already (at least nominally) protected area in north Peru: the Refugio de Vida Silvestre Laquipampa (D.S. N°045-2006-AG). The Pacific coast and Chiclayo, the fourth biggest city of Peru, are only 50–60 km away in a straight line (Fig. 1).

The surprising discovery of a conspicuous macrophyte in this area, reveals that there is significant undersampling, even in readily accessible, recognised biodiversity-rich areas of northern Peru. The lack of knowledge of biodiversity may have dire consequences for its survival as recent incidents tragically demonstrate. Contrary to our expectations, governmental monitoring and protection – given the relative proximity to major cities – could not prevent a series of wildfires in several protected areas in northern Peru, as a result of poor agricultural burning practices during a drought in 2016 (Novoa and Finer 2017). The Laquipampa Wildlife Refuge suffered a major wildfire in the “La Pescadera” sector in November 2016, with a reported loss of 20 hectares of protected forest and additional 70 hectares of buffer forest (La República, Nov/18/2016). The fires mostly raged in areas covered with dry, scrub vegetation some 5.5 km away from the much more humid, shady and less seasonal habitat in which the new *Nasa* is found.

Although detailed information on the extent of the damage varies, it is clear that even areas within official conservation programmes remain virtually unprotected when it comes to drought-promoted and human-induced wildfires.

The habitat, to which the new species is endemic, lies well in the Amotape-Huancabamba Zone (AHZ) in southern Ecuador and large parts of northern Peru (for details see: Weigend 2002, 2004). This floristic region is an important centre of biodiversity and a hotspot of endemism (Berry 1982, Ayers 1999, Weigend 2002) as repeatedly demonstrated for several plant groups (Weigend et al. 2005; Struwe et al. 2009; Deanna et al. 2018) and, in particular, for Loasaceae (Dostert and Weigend 1999; Weigend 2004; Henning and Weigend 2009a). The new taxon described here underlines the importance of this region in terms of phytodiversity and showcases the exceptional morphological divergence even between closely allied taxa in this region.

The traditional subdivision of *Nasa* into four Series (*Alatae*, *Carunculatae*, *Grandiflorae* and *Saccatae* – Urban and Gilg 1900) remains useful to coarsely characterise the morphology, growth form and appearance of a species; however these divisions do not seem to agree with clades retrieved by molecular studies (Weigend et al. 2004; Weigend and Gottschling 2006). *Nasa* series *Saccatae*, to which the taxon here described would have been assigned, has turned out to be a highly unnatural group defined on the basis of plesiomorphic characters (annual to subperennial herbs with tilt revolver flowers and contrastingly coloured nectar scales with small, erect apical wings: Weigend 1997; Weigend and Gottschling 2006). Ongoing molecular work has, however, repeatedly retrieved some monophyletic clades within the polyphyletic *Saccatae*, namely the *N.stuebeliana* (Urb. & Gilg) Weigend, *N.poissoniana* (Urb. & Gilg) Weigend and *N.triphylla* (Juss.) Weigend groups (Weigend and Gottschling 2006; Acuña et al. in prep.). The latter, although recently revised using copious herbarium material (Dostert and Weigend 1999), has already been subject to a taxonomic expansion (Henning and Weigend 2009a). This is not surprising, as it is one of the most widespread clades, present from northern Peru in the south to northern Venezuela (Nasatriphyllasubsp.papaverifolia (Kunth) Weigend) and Mexico (*Nasa* ‘*triphylla*’ subsp. rudis (Benth.) Weigend) in the north. Most of the taxa (16 out of 22 species and subspecies) have been found in the Amotape-Huancabamba zone (Dostert and Weigend 1999; Henning and Weigend 2009a). Typically, one or two taxa of the *Nasatriphylla* species group grow in each relic forest of the western slopes of the AHZ Andes (Weigend 2002). For example, Nasahumboldtiana(Urb. & Gilg)Weigendsubsp.glandulifera T.Henning & Weigend and N.humboldtianasubsp.subtrifoliata T.Henning & Weigend, were described from the opposite margins of the so-called “Bosque de Kañaris”, the largest of these forest fragments, to northeast to the Refugio de Vida Silvestre Laquipampa (Henning and Weigend 2009a). NasapteridophyllaWeigend & Dostertsubsp.geniculata Weigend & Dostert and N.humboldtianasubsp.obliqua Dostert & Weigend, are endemic to the “Bosque de Monte Seco”, another neighbouring relic forest, towards the southeast, in the Department Cajamarca (Dostert and Weigend 1999).

## Materials and methods

### Collection locality

The Refugio de Vida Silvestre Laquipampa was established in 2006 and spans an area of 8330 hectares on the western slope of the Cordillera Occidental between 500 and 2500 m a.s.l. It is located in the Department Lambayeque, Province Ferreñafe close to the border with Department Cajamarca. The reserve is mainly covered by seasonally dry tropical forest, with increasingly humid conditions towards the mountain ridges (SERNANP 2018http://www.sernanp.gob.pe/laquipampa).

### Plant material

The material studied was obtained from the collection locality and is preserved in HUT (Thiers 2018). Stereomicroscopes and light microscopes were used for its study.

### Molecular methods

Total DNA was extracted from silica gel or herbarium preserved material of 68 species and subspecies of Cornales, using the CTAB method (Doyle and Doyle 1987). We analysed sequences from four plastid regions: *trn*L–*trn*F, *mat*K, the *trn*S–*trn*G intergenic spacers and the *rps*16 intron. These have proved to be informative to infer the phylogenetic relationships in Loasoideae (Weigend et al. 2004; Hufford et al. 2005; Weigend and Gottschling 2006; Acuña et al. 2017). Sequences were newly generated for this study or for previously published research by our working group (Acuña et al. 2017, 2018; Henning et al. 2018). These were combined in a single matrix with 4 partitions. There is full overlap for the markers except *mat*K, that was not amplified for *Nasaformosissima* Weigend and *Cevalliasinuata* Lag. The partial *mat*K sequence of the latter was obtained from GenBank. (Hufford et al. 2005). Outgroups were selected based on Xiang et al. (2011) and APGIV (2016). The species *Cornusperuviana* J.F.Macbr., *Fendlerarupicola* Engelm. & A.Gray, *Hydrangeaoerstedii* Briq. and *Nyssatalamancana* Hammel & N.Zamora were selected as distantly related outgroups. The respective GenBank accession numbers for all sequences are shown in the Suppl. material 1. Amplification, sequencing and alignment protocols followed Acuña et al. (2017).

Phylogenetic reconstructions were carried out, employing Maximum Likelihood (ML) in RAxML v. 8.1.X (Stamatakis 2014), included in RAxMLGUI v. 1.5b1 (Silvestro and Michalak 2012) and Bayesian Inference (BI) in MrBayes 3.2.6 (Huelsenbeck and Ronquist 2001) in the CIPRES Science Gateway (Miller et al. 2010). Based on the Akaike information criterion, FindModel (source: http://hcv.lanl.gov/content/sequence/findmodel/findmodel.html), which implements Posada and Crandall’s (2001) Modeltest, selected GTR+Γ as the substitution model that fits the nucleotide dataset. Following Xiang et al. (2011), *Cornusperuviana* was chosen to root the trees. The statistical support for the nodes was assessed by 1000 ML thorough bootstrap replicates in 100 runs. The BI analyses were conducted in four independent runs with one cold and three heated chains; the Markov chain had a length of 10 million generations, sampled every 1000 generations. After convergence was assessed in Tracer 1.5 (Rambaut and Drummond 2014), the first 2.5 million generations were discarded as burn-in.

## Results

### Taxonomic treatment

#### Nasa angeldiazioides

Taxon classificationPlantaeCornalesLoasaceae

T.Henning, R.H.Acuña, E.Rodr., L.García-Llatas & Weigend sp. nov.

urn:lsid:ipni.org:names:60478524-2

Figs 2A–E , 3

##### Type.

Peru: Dept. Lambayeque, Provincia Ferreñafe, Distrito Incahuasi, “Refugio de Vida Silvestre Laquipampa”, ruta Piedra Parada, April 2015, *L. F. García Llatas 333* (Holotype: HUT, Isotypes: HUT)

##### Diagnosis.

*Nasaangeldiazioides* is similar to *N.bicornuta*, *N.pteridophylla* and *N.urens* but differs in having strongly amplexicaul leaves, sessile to amplexicaul prophylls on the pedicels and flowers with white petals and dark red nectar scales. The unique interrupted bipinnatisect leaves with rounded leaflet apices distinguish *N.angeldiazioides* from all other taxa of *Nasa* and Loasaceae as a whole.

##### Description.

Annual herb 30–50 (–110) cm tall. Stem subterete to weakly grooved, 3–9 mm thick at base, pale green with dispersed darker green streaks and dots and whitish protuberances; set with scattered yellowish setae 1–1.5 mm long and covered with medifixed t-shaped hairs, 0.1–0.3 mm. Adventitious roots present in the L-shaped stem base. Leaves alternate, petiolate below (petiole to 10–15 mm), amplexicaul above sometimes with decurrent base, sessile in between; glabrescent; lamina oblong to widely rhomboidal in outline, (80-) 150–280 (-310) mm long and (25-) 60–220 (-280) mm wide (the petiolate leaves smaller than the sessile ones), pinnate at the base and apex, bipinnatisect in the central part, dissected nearly to midvein, central pinnae subpinnatisect, lobules narrowly-oblong; apex acuminate; abaxial surface covered with short, prostrate, yellowish glochidiate hairs, 0.05–0.1 mm and scabrid hairs 0.2–0.3(-0.5) mm along the veins; adaxial surface covered with few scabrid hairs 0.2–0.3 mm long (never on the veins), venation pinnate. Inflorescences of 2–5 terminal or axillary monochasial branches each 10–20 cm long, with (3-) 5–10 (-15) pendent flowers per branch. Flowers borne opposite to an amplexicaul upper leaf, bracts sometimes recaulescent, simple, ovate, margin dentate, sessile, (10-) 15–30 (-33) long and (6-) 10–17 (-25) mm wide, pedicel often provided with a single, ovate, sessile to amplexicaul prophyll, (15-) 20–25 mm long and 12–15 mm wide. Flowers pentamerous, pedicels 30–50 mm, green at the upper half, basally brownish, appearing dessicated, calyx covered densely with scabrid hairs (0.5–0.7 mm) and sparse glochidiate hairs (0.1–0.2 mm), tube conical, 4 × 3 mm, calyx lobes ovate acuminate, 7 × 4 mm, densely covered with scabrid hairs on back. Petals spreading to slightly reflexed, white, deeply cymbiform, cucullate, 15–23 mm long, 5 mm wide and 9–10 mm deep, base green, unguiculate and abruptly widened into two small triangular teeth 2 mm from base, these bent towards the centre of the petal, almost touching each other and leaving only a narrow gap for the stamens, adaxial surface set with scattered scabrid (0.2–0.3 mm) and uniseriate short glandular (3–7 cells) (0.3–0.7 mm) hairs; abaxial surface set with scattered glochidiate (0.1–0.2 mm) and uniseriate short glandular (3–7 cells) (0.3–0.7 mm) hairs. Nectar scales dark red at the base (sacs), white towards the neck and wings, with triangular back, much narrowed above, 7–8 × 4 mm, base incurved, back with two conspicuous, globose nectar sacs about 2 mm in diameter, scale back with 4–5 transversal calli, neck thickened and slightly recurved, laterally protracted into two small erect wings 1 mm long and 0.5 mm wide. Staminodia 9–10 mm long, base slightly dilate, filiform above, papillose, yellowish. Stamens in epipetalous fascicles of 12–15 each, filaments 8–10 mm, white, anthers 0.3 mm long and wide, yellow. Ovary inferior, with three parietal placentae and numerous ovules. Fruit a capsule, horizontal to semi-erect due to the sigmoid pedicel that elongates postflorally, capsule narrowly clavate, slightly curved, purplish, opening with 3 apical valves.

## Discussion

### Affinities

*Nasaangeldiazioides* adds even more morphological diversity to highly diverse *Nasa*. In previous taxonomic works (Weigend et al. 1998; Weigend 2004; Henning and Weigend 2009b, 2011; Henning et al. 2011), the enormous variability in almost all aspects of plant morphology found in this genus has been demonstrated. *Nasa* has evolved an incredible range of characteristics in concert with habitat-exploitation and pollinator recruitment, including different life history, growth form, leaf shape, indumentum, inflorescence morphology and floral architecture characters. As a result, major clades within *Nasa* have few or no evident morphological apomorphies, but are rather defined by unique combinations of characters that individually can also occur in other, distantly related taxa. The assignment of species to one of these groups is usually possible, based on morphological traits, especially regarding growth form, phyllotaxis, leaf blade division and details of flower morphology. The species described here, however, presents some challenges in this regard and an allocation to a major clade would have been tentative without molecular evidence.

**Figure 2.**
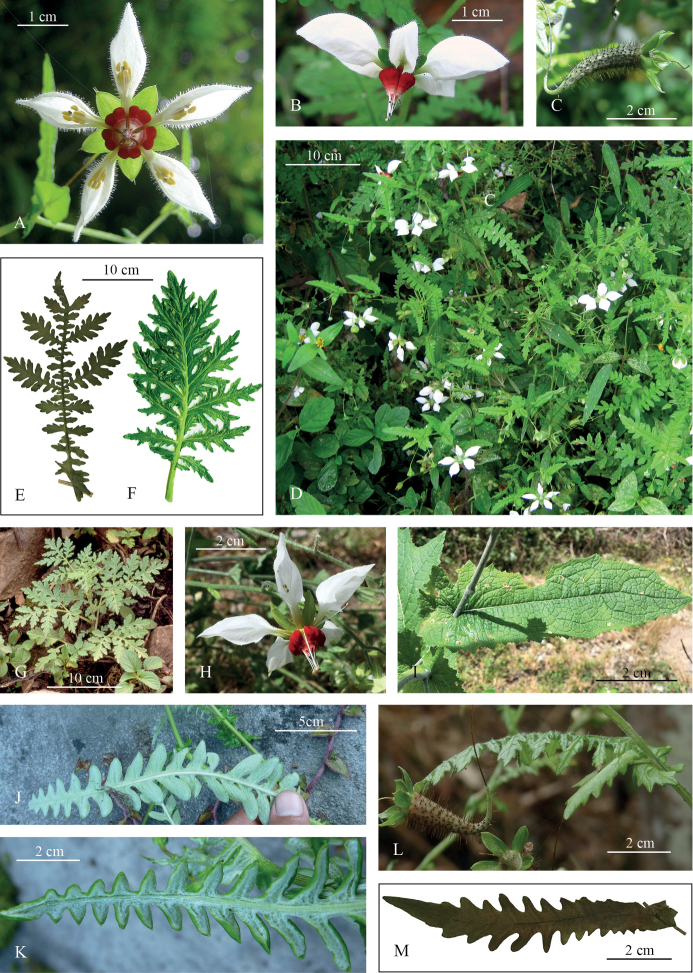
Morphology of *Nasaangeldiazioides* (**A–E, L, M**) and similar taxa (**F–K**). **A** Flower, frontal view **B** Flower, lateral view **C** Capsule, lateral view, note the thin, curved pedicel **D** General habit of several flowering plants in their natural habitat, Bosque de Chiñama **E** Mature basal leaf **F** Mature leaf of *Nasaurens***G** Young plant of *N.pteridophylla***H** Flower of *Nasaformosissima*, lateral view **I** Amplexicaul bract of *N.formosissima***J, K** Leaves of *Angeldiaziaweigendii*, abaxial surface, note the overall shape and the ampexicaul leafbase visible in the background **L** Bract, prophyll and capsules of *Nasaangeldiazioides***M** Lowermost bract of *N.angeldiazioides*, outlined from a specimen photograph. (Credits: photographs **A, B, D** B. Esquerre-Ibañez **J, K** Mario Zapata).

**Figure 3.**
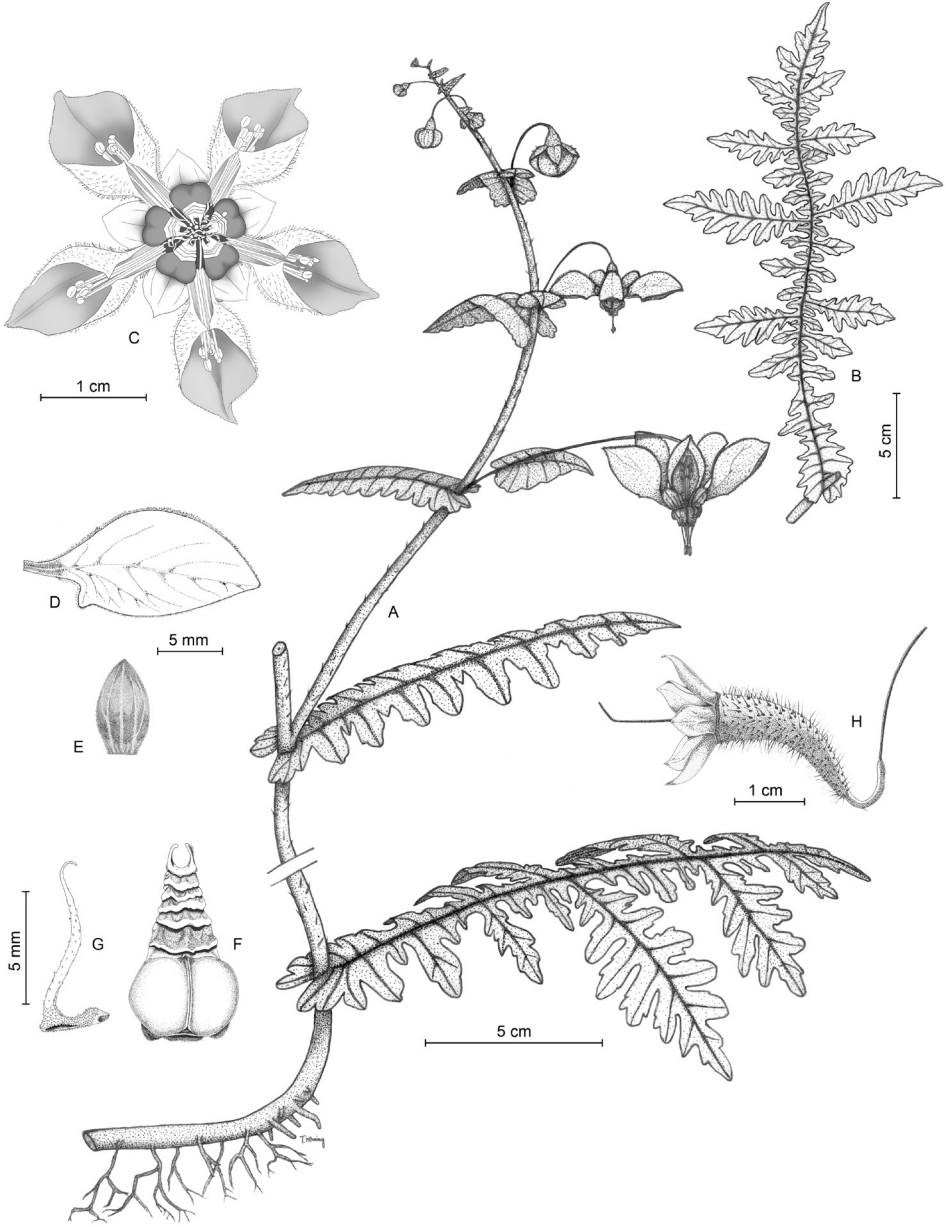
*Nasaangeldiazioides*. **A** Flowering shoot **B** Mature leaf **C** Flower **D** Petal **E** Calyx lobe **F** Nectar scale, abaxial view **G** Staminode, lateral view **H** Capsule, lateral view. Drawing prepared by L. García (**D–H**), T. Henning (**A, B**) and R. Acuña (**C**).

*Nasaangeldiazioides* shares the presence of amplexicaul bracts, with several species of the *Nasastuebeliana* group (e.g. *N.formosissima* Fig. 2H, I, Weigend and Rodríguez 2003), but also with individual taxa of other clades. The white petals in combination with dark red nectar scales (Fig. 2A, B) are found in a range of “Saccatae” species, sometimes in combination with a narrowed neck and several transversal calli, e.g. in *Nasapicta*, a taxonomically isolated but geographically widespread species in northern Peru, sister to the compound leaved *Nasavenezuelensis* (Steyerm.) Weigend + *Nasatriphylla* groups (Weigend and Gottschling 2006, this study). Superficially similar deeply dissected leaves are found in *Nasaurens* (Fig. 2F), but the pattern of leaf subdivision is more similar to what is found in species of the *Nasatriphylla* group. Overall, the thin, angular pedicels (esp. in fruit (Figs 2C, 3A), clavate capsules (Figs 2C, 3H) and especially the deeply divided leaves (Figs 2E and 3B) indicate a close affinity to *Nasapteridophylla* (Fig. 2G) and *Nasabicornuta*. Despite the close overall similarity, the peculiar type of leaf subdivision with interrupted bipinnatisect leaves with rounded leaflet apices is unique in *Nasa* and indeed the entire Loasaceae.

### Molecular placement

**Figure 4.**
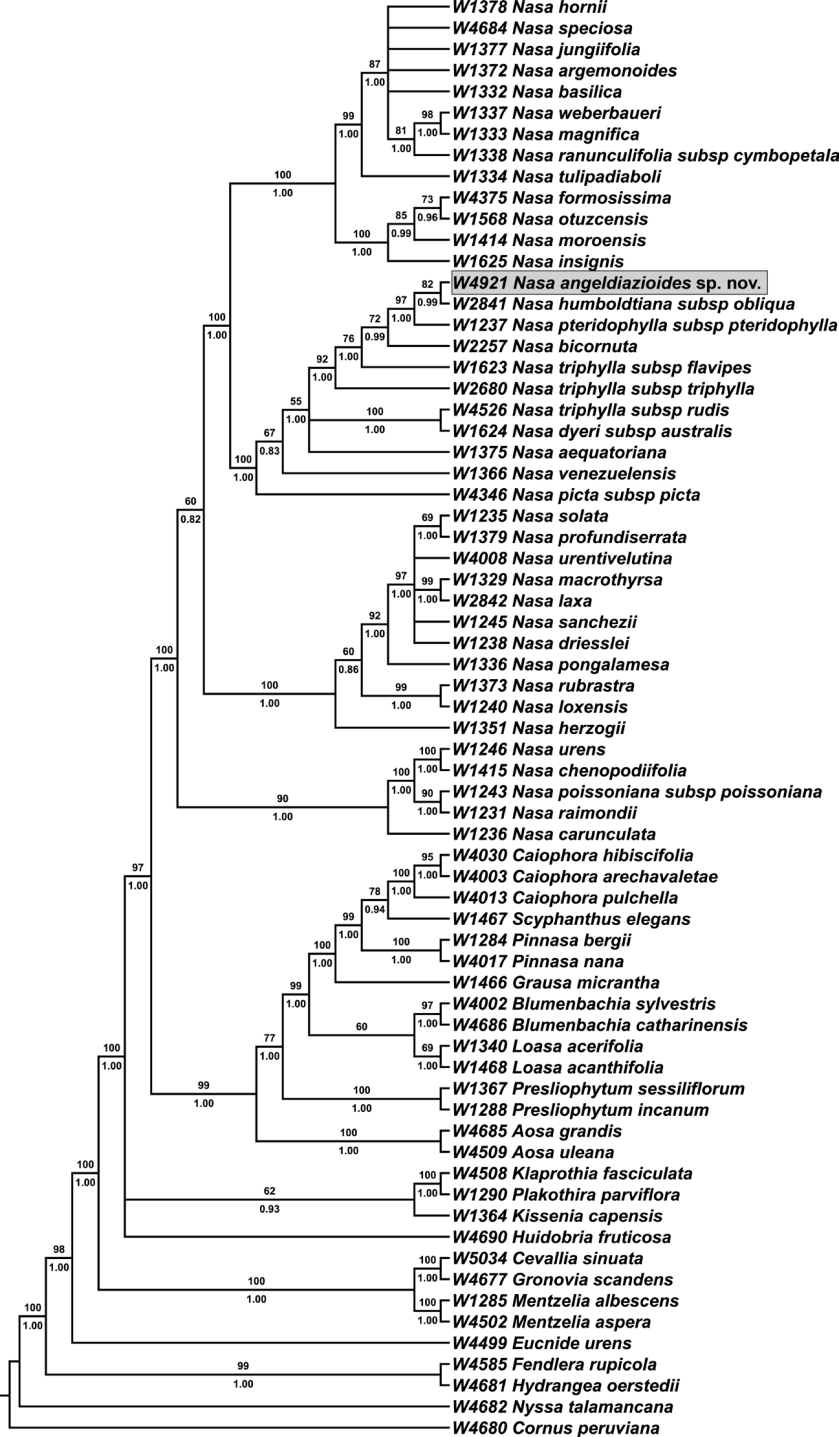
Maximum likelihood tree based on a plastid combined dataset (*mat*K, *rps*16, *trn*L-*trn*F, *trn*S-*trn*G). ML bootstrap support values are indicated above branches and Bayesian posterior probabilities are indicated below; only values above 50 and 0.5, respectively are shown. *Nasaangeldiazioides* is marked by a grey rectangle.

Plastid molecular data, clearly show that this new species belongs to the *Nasatriphylla* group and is closely related to *Nasapteridophylla* and *N.humboldtiana* (Fig. 4). Morphological and ecological similarities also seem to confirm that these three taxa are phylogenetically very close. The new species, like *Nasapteridophylla*, has ovate-acuminate sepals and semi-erect capsules, it is found on the Pacific slope of the Amotape Huancabamba Zone and grows in the understorey of low elevation, seasonally dry forests (Dostert and Weigend 1999). Most importantly, *Nasapteridophylla*, *N.humboldtiana* and the new species, all have very characteristic medifixed t-shaped trichomes that are, as far as we know, virtually restricted to this small clade within *Nasatriphylla* species group (cf. Henning and Weigend 2009a).

### Etymology

The epithet refers to a recently described monotypic genus of Asteraceae from the same area, *Angeldiaziaweigendii* M.O.Dillon & Zapata (Dillon and Zapata 2010). The latter species has an extremely peculiar leaf morphology: deeply pinnatisect, amplexicaulous leaves with rounded leaflet apices, i.e. leaves that in the living state look extremely similar to the upper leaves and bracts of the new species here described (Figs 2J–M, 3A).

### Phenology

The species was first reported by Santos Llatas Quiroz in May 2007 in the Bosque de Chiñama. Luis Felipe García Llatas collected the species in the Laquipampa Wildlife Refuge in March 2013 in sterile condition (specimen not deposited in a herbarium) and then in April 2015 with flowers and fruits (type collection deposited in HUT). An additional sighting from the Bosque de Chiñama by Boris Esquerre-Ibañez reported full flowering plants in June 2014 (B. Esquerre-Ibañez photographic evidence, no specimen).

The life-cycle of this taxon is strongly linked to the precipitation seasonality and its corresponding inter-annual variation. Annual plant development is mostly affected by the amount of precipitation during the growing season in February and March. Flowering time coincides with other annual taxa of the group in that area, which is typically starting with the end of the rainy- and beginning of the dry season. The length of the flowering period in turn is proportional to the intensity of summer rains during the dry season and can last between some weeks to up to three months depending on overall humidity. Accordingly, fruiting plants can be found from May onwards.

### Distribution and ecology

So far, this species is known only from Laquipampa and the neighbouring Bosque de Chiñama (Fig. 1), another, smaller forest fragment towards the northeast (B. Esquerre-Ibañez, pers obsv.). All known specimens are from the type locality in the Refugio de Vida Silvestre Laquipampa (Figs 2C, L), the population from the Bosque de Chiñama is so far only documented photographically (Figs 2A, B, D). The taxon inhabits forest edges and clearings of seasonally dry forests from 1500–2000 m elevation. It can be found in shady areas, on rocky, but humus rich soils. The species may tolerate some degradation of its habitat in areas of secondary forest or roadsides, but so far has only been collected in primary forest.

The associated arboreal and shrubby species that allow the development of shady, humid microclimates and soils rich in decaying plant matter are the "Pasallo" (*Eriothecaruizii* (K. Schum.) A. Robyns, Malvaceae) that, at the time of the collections, show fresh foliage and provide shade to many herbaceous species around, as well as *Clusia* sp. (Clusiaceae), *Bauhiniaweberbaueri* Harms (Fabaceae), *Tecoma* sp. (Bignoniaceae) and “San Pedro” (*Trichocereuspachanoi* Britton & Rose, Cactaceae). The accompanying ombrophile herbaceous taxa include *Cranichis* sp. (Orchidaceae), *Callisiamonandra* (Sw.) Schult. & Schult. f. (Commelinaceae), *Dioscorea* sp. (Dioscoreaceae), *Commelina* sp. (Commelinaceae) and *Oxalis* sp. (Oxalidaceae).

No pollinator observations are available for this species, but based on flower morphology, it likely falls into the group of taxa predominantly pollinated by rather specialised short-tongued bees (Weigend and Gottschling 2006; Henning et al. 2018).

### Preliminary conservation status

*Nasaangeldiazioides* has only been reported from two relic forests in close proximity whose areas occupy less than 200 km^2^. Given the relatively easy accessibility and the comparatively good knowledge of the floristic inventory of the region in general, it is rather unlikely that vast populations have been overlooked in adjacent areas. Furthermore, this taxon is found as part of the undergrowth flora of otherwise intact primary forests. Unlike closely related taxa that are frequently found in open, disturbed situations, such as roadsides or field margins (*N.pteridophylla*, *N.bicornuta*) or in the undergrowth of secondary vegetation and for example, coffee plantations (*N.humboldtiana* subspp.), *N.angeldiazioides* seems dependent on native vegetation and is incapable of thriving in habitats that are subject to change caused by human activities. Due to its very restricted known range, small populations (L. García Llatas, B. Esquerre-Ibañez, pers. obs.) and the serious environmental threats (wildfires, deforestation, agriculture) that the whole north-western slope of the Western Cordillera of Peru faces, we consider this species as Critically Endangered (CR B1a,biv), according to the IUCN threatened species assessment guidelines (IUCN 2001, 2017).

### Additional specimens examined

Although reported twice from the Bosque de Chiñama, the taxon has so far only been collected in Laquipampa. Only the type collection from 2015 is deposited in the Herbario de la Universidad Nacional de Trujillo (Herbarium Truxillense, HUT).

## Supplementary Material

XML Treatment for Nasa angeldiazioides

## Supplementary materials

Supplementary material 1 List of taxa sampled for the molecular analyses with their respective voucher specimen (herbarium acronyms in parentheses), geographic origins and GenBank accession numbers Data type: molecular data This dataset is made available under the Open Database License (http://opendatacommons.org/licenses/odbl/1.0/). The Open Database License (ODbL) is a license agreement intended to allow users to freely share, modify, and use this Dataset while maintaining this same freedom for others, provided that the original source and author(s) are credited.Tilo Henning, Rafael Acuña Castillo, Eric Frank Rodríguez Rodríguez, Luis Felipe García Llatas, Maximilian Weigend
