# Supplementary material for: A new striking and critically endangered species of Nasa (Loasaceae, Cornales) from North Peru
Source: PhytoKeys. 2019 Apr 24;121:13–28. doi: 10.3897/phytokeys.121.33927 (PMC6494795; doi:10.3897/phytokeys.121.33927)
Supplement: Supplementary material 1 [file phytokeys-121-013-s001.pdf]

SUPPORTING INFORMATION

A new striking and critically endangered species of Nasa (Loasaceae, Cornales) from North Peru

Appendix. List of taxa sampled for the molecular analyses with their respective voucher specimen (herbarium acronyms in parentheses), geographic origins and GenBank accession numbers. Dashes (_) indicate missing data.

Taxon	Voucher	County of Origin	GenBank Codes			
			trnL-trnF	*matK*	*trnS-trnG*	*rps16*
Aosa grandis (Standl.) R.H.Acuña & Weigend	*Acuña et al. 1264* (USJ)	Costa Rica	MF972120	MF972100	MF972129	MF972110
Aosa uleana Weigend	*Joßberger 342* (BONN)	Brazil	KY286998	KY286728	KY286908	KY286818
Blumenbachia catharinensis Urb. & Gilg	*Trevisan 1723* (BONN)	Brazil	KY287006	KY286736	KY286916	KY286826
Blumenbachia sylvestris Poepp.	*Cocucci & Sérsic 4780* (CORD)	Argentina	KY286969	KY286700	KY286879	KY286789
Caiophora arechavaletae (Urb.) Urb.	*Weigend 9330* (BSB)	Brazil	KY286970	KY286701	KY286880	KY286790
Caiophora hibiscifolia (Griseb.) Urb. & Gilg	*Ackermann 1103* (BONN)	Argentina	KY286988	KY286719	KY286898	KY286808
Caiophora pulchella Urb. & Gilg	*Cocucci & Sérsic 4840* (CORD)	Argentina	KY286976	KY286707	KY286886	KY286796
Cevallia simuata Lag.	*Spencer 418* (RSA)	Mexico	MK333074	_	MK333040	MK333006
Cevallia simuata Lag.	*Waterbrook 175* (WS)	Unknown	_	AF503301	_	_
Cornus	*Acuña et al.*	Costa Rica	MK333069	MK332968	MK333035	MK333001

peruviana J.F.Macbr.	*1230* (USJ)					
Cornus peruviana J.F.Macbr.	*Xiang 02-02* (NCSC)	Costa Rica	_	_	_	_
Eucnide urens Parry ex Coville	*Weigend 9153* (BSB)	United States	KY286996	KY286726	KY286906	KY286816
Fendlera rupicola Engelm. & A.Gray	*Acuña 1220* (BONN)	Germany (cultivated)	MK333067	MK332966	MK333033	MK332999
Fendlera linearis Rehder	*Hinton et al. 16566* (US)	Unknown	_	_	_	_
Grausa micrantha (Poepp.) Weigend & R.H.Acuña	*Grau s.n.* (M)	Chile	KY286957	KY286688	KY286867	KY286777
Gronovia scandens L.	*Jiménez & Majure 2895* (USJ)	Costa Rica	MK333068	MK332967	MK333034	MK333000
Gronovia scandens L.	*Weigend et al. 8522* (BSB)	Peru	_	_	_	_
Huidobria fruticosa Phil.	*Luebert & Moreira 2991* (SGO)	Chile	MK333072	MK332971	MK333038	MK333004
Huidobria fruticosa Phil.	*Dillon 8034* (F)	Chile	_	_	_	_
Hydrangea oerstedii Briq.	*Acuña et al. 1231* (USJ)	Costa Rica	MK333070	MK332969	MK333036	MK333002
Hydrangea seemannii x *peruviana*.	*Goetghebeur 12381* (GENT)	Belgium (cultivated)	_	_	_	_
Kissenia capensis Endl.	*Greuter 21627* (B)	South Africa	KY286944	KY286675	KY286854	KY286764
Klaprothia fasciculata (C.Presl) Poston	*Weigend et al. 7553* (B)	Peru	MK333066	MK332965	MK333032	MK332998

Klaprothia fasciculata (C.Presl) Poston	*Weigend et al. 5362* (BSB)	Peru	_	_	_	_
Loasa acanthifolia Desr.	*Weigend et al. 6924* (M)	Argentina	KY286959	KY286690	KY286869	KY286779
Loasa acerifolia Dombey ex Juss.	*Weigend et al. 6848* (M)	Argentina	KY286937	KY286669	KY286847	KY286757
Mentzelia albescens (Gillies ex Arn.) Benth. & Hook.f. ex Griseb.	*Weigend et al. 6865* (BSB)	Argentina	KY286921	KY286653	KY286831	KY286741
Mentzelia aspera L.	*Weigend et al. 8421* (B)	Peru	MF972116	MF972096	MF972125	MF972106
Nasa aequatoriana (Urb. & Gilg) Weigend	*Weigend & Jaramillo 3937* (F)	Ecuador	KY286947	KY286678	KY286857	KY286767
Nasa argemonoides (Juss.) Weigend	*Weigend s.n.* (M)	Colombia	MK333053	MK332954	MK333019	MK332985
Nasa basilica T.Henning & Weigend	*Weigend & al. 97/370* (F)	Peru	KY286935	KY286667	KY286845	KY286755
Nasa bicornuta (Weigend) Weigend	*Weigend et al. 8600* (MO)	Peru	MK333060	MK332960	MK333026	MK332992
Nasa carunculata (Urb. & Gilg) Weigend	*Weigend & Weigend 2000/363* (HUSA)	Peru	MK333042	MK332942	MK333008	MK332974
Nasa chenopodiifolia (Desr.) Weigend	*Weigend et al. 7685* (M)	Peru	MF972162	MF972134	MF972176	MF972148

Nasa driesslei Weigend	*Henning & Schneider 243* (BSB)	Peru	KY286917	KY286649	KY286827	KY286737
Nasa dyeri subsp. *australis* Dostert & Weigend	*Dostert 98/80* (MSB)	Peru	MF972165	MF972137	MF972179	MF972151
Nasa formosissima Weigend	*Henning & Brokamp 13* (B)	Peru	MK333065	_	MK333031	MK332997
Nasa herzogii (Urb. & Gilg) Weigend	*Müller & Heinrichs 6596* (LPB)	Bolivia	MK333051	MK332952	MK333017	MK332983
Nasa hornii (Weigend) Weigend	*Weigend & Horn 3815* (M)	Ecuador	MK333056	MK332956	MK333022	MK332988
Nasa humboldtiana subsp. *obliqua* Dostert & Weigend	*Henning & Schulz 40* (BSB)	Peru	MK333061	MK332961	MK333027	MK332993
Nasa humboldtiana subsp. *obliqua* Dostert & Weigend	*Dostert 98/154* (M)	Peru	_	_	_	_
Nasa insignis Weigend & E.Rodr.	*Weigend et al. 7563* (M)	Peru	MK333059	MK332959	MK333025	MK332991
Nasa insignis Weigend & E.Rodr.	*Dostert 98/161* (M)	Peru	_	_	_	_
Nasa jungiifolia (Weigend) Weigend	*Weigend & Horn 3838* (M)	Ecuador	MK333055	MK332948	MK333021	MK332987
Nasa laxa (J.F.Macbr.) Weigend	*Henning & Schulz 29* (B)	Peru	MK333062	MK332962	MK333028	MK332994

Nasa laxa (J.F.Macbr.) Weigend	*Weigend et al. 98/547* (F)	Peru	_	_	_	_
Nasa loxensis (Kunth) Weigend	*Grant & Struwe 01-4063* (BSB)	Ecuador	MK333044	MK332944	MK333010	MK332976
Nasa macrothyrsa (Urb. & Gilg) Weigend	*Weigend et al. 97/s.n.* (M)	Peru	KY286934	KY286666	KY286844	KY286754
Nasa magnifica (Urb. & Gilg) Weigend	*Weigend et al. 97/468* (F)	Peru	MK333047	MK332949	MK333013	MK332979
Nasa moroensis Weigend	*Weigend 7694* (B)	Peru	MF972161	MF972133	MF972175	MF972147
Nasa otuzcensis Weigend & E.Rodr.	*Rodríguez et al. 2374* (HUT)	Peru	MK333058	MK332958	MK333024	MK332990
Nasa picta (Hook.) Molinari	*Henning & Schulz 33* (BSB)	Peru	MK333064	MK332964	MK333030	MK332996
Nasa picta (Hook.) Molinari	*Henning & Schneider 2* (B)	Peru	_	_	_	_
Nasa poissoniana subsp. *poissoniana* (Urb. & Gilg) Weigend	*Weigend & Weigend 00/208* (NY)	Peru	KY286918	KY286650	KY286828	KY286738
Nasa pongalamesa Weigend	*Weigend et al. 2000/752* (BSB)	Peru	MF972114	MF972094	MF972123	MF972104
Nasa profundiserrata Weigend	*Weigend et al. 3626* (M)	Colombia	MK333057	MK332957	MK333023	MK332989
Nasa pteridophylla	*Weigend et al. 97/307C* (M)	Peru	MK333043	MK332943	MK333009	MK332975

subsp. *pteridophylla* Weigend & Dostert						
Nasa raimondii (Standl. & F.A.Barkley) Weigend	*Weigend & Weigend 2000/289* (HUSA)	Peru	MF972113	MF972093	MF972122	MF972103
Nasa ranunculifolia subsp. *cymbopetala* (Urb. & Gilg) Weigend	*Weigend et al. 97/466* (F)	Peru	MK333050	MK332951	MK333016	MK332982
Nasa rubrastra (Weigend) Weigend	*Schwerdtfeger 22207* (GOET)	Ecuador	MK333054	MK332955	MK333020	MK332986
Nasa sanchezii T.Henning & Weigend	*Henning & Schneider 242* (B)	Peru	MK333045	MK332945	MK333011	MK332977
Nasa solata (J.F.Macbr.) Weigend	*Weigend & Dostert 98/259* (M)	Peru	MK333041	MK332941	MK333007	MK332973
Nasa sp Laquipampa.	*García 333* (HUT)	Peru	MK333073	MK332972	MK333039	MK333005
Nasa speciosa (Donn.Sm.) Weigend	*Acuña et al. 1261* (USJ)	Costa Rica	MF972119	MF972099	MF972128	MF972109
Nasa triphylla subsp. *flavipes* Weigend & Dostert	*Weigend & Dostert 98/203* (M)	Peru	MF972164	MF972136	MF972178	MF972150
Nasa triphylla subsp. *rudis* (Benth.) Weigend	*Acuña et al. 1211* (BONN)	Costa Rica	MF972117	MF972097	MF972126	MF972107
Nasa triphylla subsp. *triphylla*	*Weigend & Brokamp 9098*	Ecuador	MF972170	MF972142	MF972184	MF972156

(Juss.) Weigend	(B)					
Nasa tulipadiaboli T.Henning & Weigend	*Weigend et al. 01/443* (B)	Peru	MK333048	MK332947	MK333014	MK332980
Nasa urens (Jacq.) Weigend	*Weigend & Förther 97/542* (USM)	Peru	MK333046	MK332946	MK333012	MK332978
Nasa urentivelutina Weigend	*Henning & Brokamp 9* (B)	Peru	MK333063	MK332963	MK333029	MK332995
Nasa venezuelensis (Steyerm.) Weigend	*Weigend 3604* (M)	Venezuela	MK333052	MK332953	MK333018	MK332984
Nasa weberbaueri (Urb. & Gilg) Weigend	*Weigend & Dostert 98/261* (F)	Peru	MK333049	MK332950	MK333015	MK332981
Nyssa sylvatica Marshall	*ZJW374-9* (Unknown)	United States (cultivated)	_	_	_	_
Nyssa talamancana Hammel & N.Zamora	*Acuña et al. 1237* (USJ)	Costa Rica	MK333071	MK332970	MK333037	MK333003
Pinnasa bergii (Hieron.) Weigend & R.H.Acuña	*Weigend 6800* (BSB)	Argentina	KY286920	KY286652	KY286830	KY286740
Pinnasa nana Phil.	*Weigend et al. 7080* (BSB)	Argentina	KY286980	KY286711	KY286890	KY286800
Plakothira parviflora J.Florence	*Weigend s.n.* (BSB)	France (Marquesas Islands)	KY286926	KY286658	KY286836	KY286746
Presliophytum incanum (Graham) Weigend	*Weigend & Förther 97/848* (F)	Peru	KY286924	KY286656	KY286834	KY286744

Presliophytum sessiliflorum (Phil.) R.H.Acuña & Weigend	Ehrhardt s.n.(M)	Chile	KY286945	KY286676	KY286855	KY286765
Scyphanthus elegans Sweet	*Grau & Ehrhardt 2-093* (M)	Chile	KY286958	KY286689	KY286868	KY286778
